# Latent profiles of workplace violence exposure and predictive modeling of sleep disorders among emergency department nurses: A nationwide cross-sectional study

**DOI:** 10.1097/MD.0000000000050549

**Published:** 2026-09-04

**Authors:** Qingli Chen, Mei Yuan, Wei Zhang, Le Tong

**Affiliations:** a Department of Emergency Medicine, West China Hospital, Sichuan University, Chengdu, Sichuan, China; b West China School of Nursing, Sichuan University, Chengdu, Sichuan, China; c Institute of Disaster Medicine, Sichuan University, Chengdu, Sichuan, China; d Nursing Key Laboratory of Sichuan Province, Chengdu, Sichuan, China.

**Keywords:** emergency nurses, latent profile analysis, machine learning, sleep disorders, workplace violence

## Abstract

Workplace violence and sleep disorders are common among emergency department (ED) nurses, yet heterogeneity in violence exposure and its relationship with sleep disorders remains poorly understood. This nationwide cross-sectional study included 1540 Chinese ED nurses. Latent profile analysis identified violence exposure patterns, while LASSO, 7 machine learning algorithms, and SHAP were used for sleep disorder prediction and model interpretation. Three violence profiles were identified: low-level (87.0%), moderate multi-form (10.3%), and high multi-form exposure (2.7%). Sleep disorders affected 59.3% of participants. Random forest showed the highest predictive performance (ROC AUC = 0.624). SHAP analysis showed greater predictive contributions from emotional exhaustion, cynicism, and occupational characteristics than from violence exposure profiles. Workplace violence exposure among ED nurses was heterogeneous. Burnout-related and occupational characteristics provided stronger predictive information for sleep disorder status than violence profiles. External validation and longitudinal studies are needed to assess generalizability and temporal relationships.

## 1. Introduction

Workplace violence remains a persistent occupational hazard in healthcare, with consequences that reach well beyond the immediate clinical encounter. It has been defined by the World Health Organization as work-related abuse, threat, or assault – including events occurring during commuting – that explicitly or implicitly places workers’ safety, well-being, or health at risk; both physical and psychological violence are encompassed within this definition.^[1]^ Worldwide, approximately 61.9% of healthcare workers have reported exposure to workplace violence, and nurses appear to experience a higher burden than physicians.^[2]^ Emergency departments are particularly susceptible to such events, given the convergence of acute illness, time-pressured care, uncertain waiting times, family distress, and intensive nurse–patient communication demands.^[3,4]^ For emergency department nurses, who often remain in closest and most continuous contact with patients and families, this exposure is a routine occupational vulnerability.

The impact of workplace violence is not confined to immediate injury. Prior studies have associated workplace violence with anxiety, depression, post-traumatic stress disorder, reduced work efficiency, turnover intention, suicide risk, and compromised patient safety.^[5-9]^ Links with chronic physical conditions, including cardiovascular disease and type 2 diabetes, have also been reported.^[10,11]^

Sleep disorders, meanwhile, constitute another prominent occupational health problem in emergency nursing. Heavy workload, night shifts, rotating schedules, emotional strain, and repeated exposure to stressful events may disturb circadian rhythm and sleep quality.^[12,13]^ In nurses who have experienced workplace violence, sustained vigilance, emotional exhaustion, and occupational burnout may add another layer of sleep-related vulnerability.^[14,15]^

Sleep disorders are also shaped by a combination of demographic, occupational, and psychological factors, including workload, night shift work, income, workplace violence exposure, and burnout-related indicators.^[16]^ Current evidence remains limited in explaining how these factors jointly contribute to sleep disorder risk among emergency department nurses.^[3,14,17]^ Against this background, the present study focused on emergency department nurses in China, aiming to identify distinct patterns of workplace violence exposure, compare characteristics between nurses with and without sleep disorders, and examine the contribution of multidimensional factors to sleep disorder risk identification. The findings are expected to inform occupational health assessment, workplace violence prevention, and early intervention for sleep disorders in emergency nursing settings.

## 2. Materials and methods

### 2.1. Source of data

**Figure 1.**
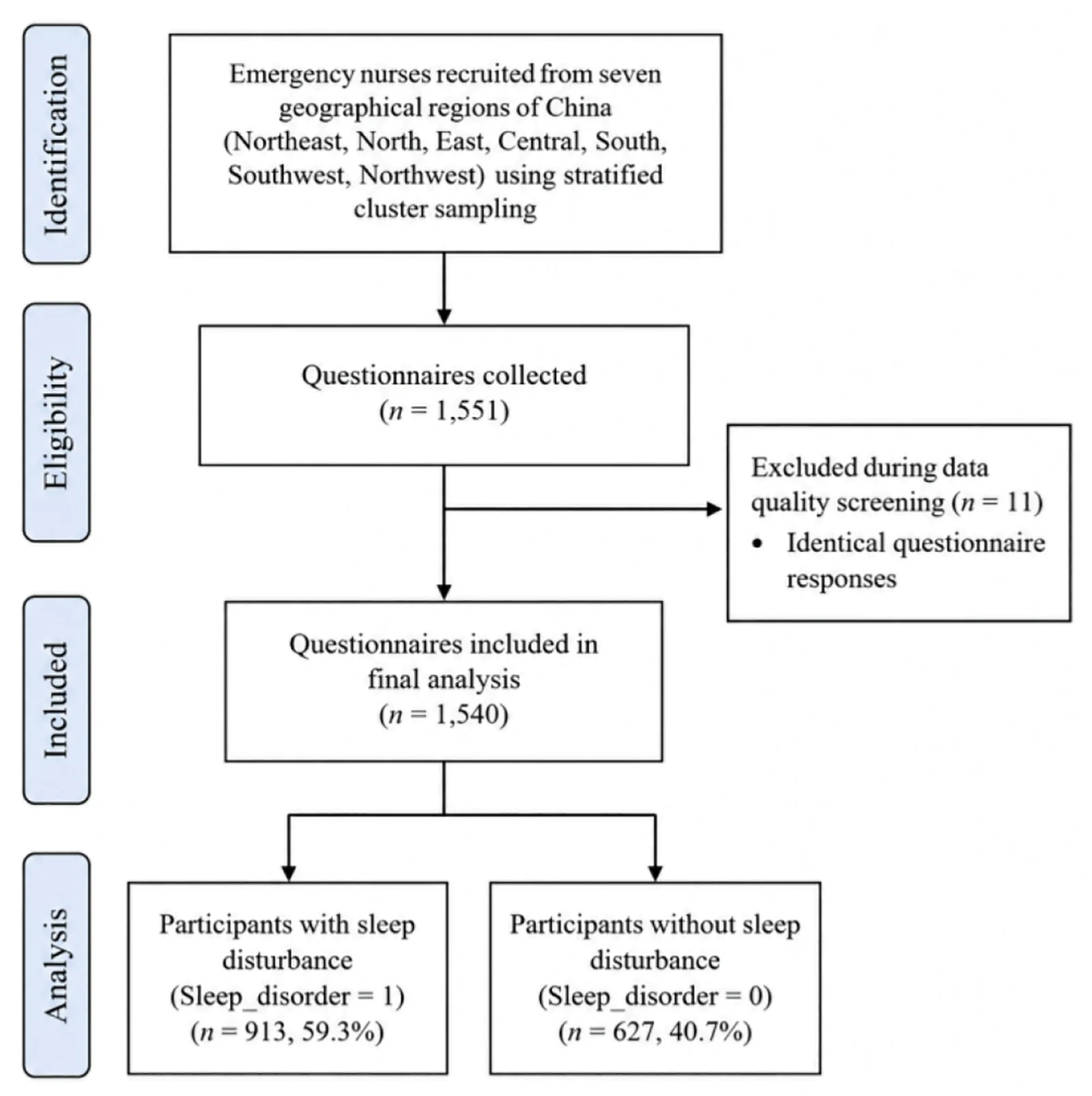
STROBE-style flow diagram of participant recruitment and inclusion. STROBE = Strengthening the Reporting of Observational Studies in Epidemiology.

A nationwide cross-sectional survey was conducted between December 26, 2023, and January 18, 2024. Emergency department nurses were recruited using stratified cluster sampling across 7 geographic regions of China: Northeast, North, East, Central, South, Southwest, and Northwest China. Eligible participants were aged ≥ 18 years and had at least 1 year of emergency nursing experience. Nurses were excluded if they were visiting nurses, were absent from work during the survey period, reported a history of psychiatric illness, or had taken psychotropic medication during the week preceding the survey. A total of 1551 questionnaires were collected. After data quality screening, 11 questionnaires with identical responses were excluded, leaving 1540 participants for the final analysis (see Fig. 1).

### 2.2. Data processing

Data were collected through an anonymous web-based questionnaire after informed consent was obtained. A trained coordinator in each hospital provided standardized instructions. All items were mandatory, and each IP address was limited to 1 submission to reduce missing and duplicate responses.

The questionnaire covered demographic and occupational characteristics, workplace violence, work-related factors, and health status. Workplace violence was assessed using 4 items adapted from the 2005 National Survey of the Work and Health of Nurses,^[18,19]^ covering psychological and physical violence perpetrated by patients/family members and leaders/colleagues during the past year. Each item was coded as a 7-level numerical frequency score ranging from 0 to 6, with higher scores indicating greater exposure frequency. The Cronbach α was 0.71.

Sleep disorder status during the previous month was assessed using the Self-administered Sleep Questionnaire (SSQ), which evaluates sleep onset latency, sleep maintenance, and early morning awakening. Each item is scored from 0 to 4, with higher scores indicating greater symptom severity. Participants were classified as screening positive if they met at least one of the following criteria: sleep latency of more than 30 minutes, difficulty resuming sleep after nocturnal awakening more than once per week, or early morning awakening with difficulty resuming sleep more than once per week. Participants meeting at least 1 criterion were coded as Sleep_disorder = 1, whereas those meeting none were coded as Sleep_disorder = 0. The SSQ showed good internal consistency (Cronbach α = 0.809). The details shown in Table S2, Supplemental Digital Content 1.

### 2.3. Statistical analysis

Latent profile analysis was conducted in Mplus 8.3 using the 4 workplace violence items as observed indicators. One- to 4-profile models were compared using AIC, BIC, aBIC, entropy, the Lo–Mendell–Rubin adjusted likelihood ratio test, and the bootstrap likelihood ratio test.^[20]^ The optimal solution was selected based on model fit, classification quality, parsimony, interpretability, and profile size.

**Figure 2.**
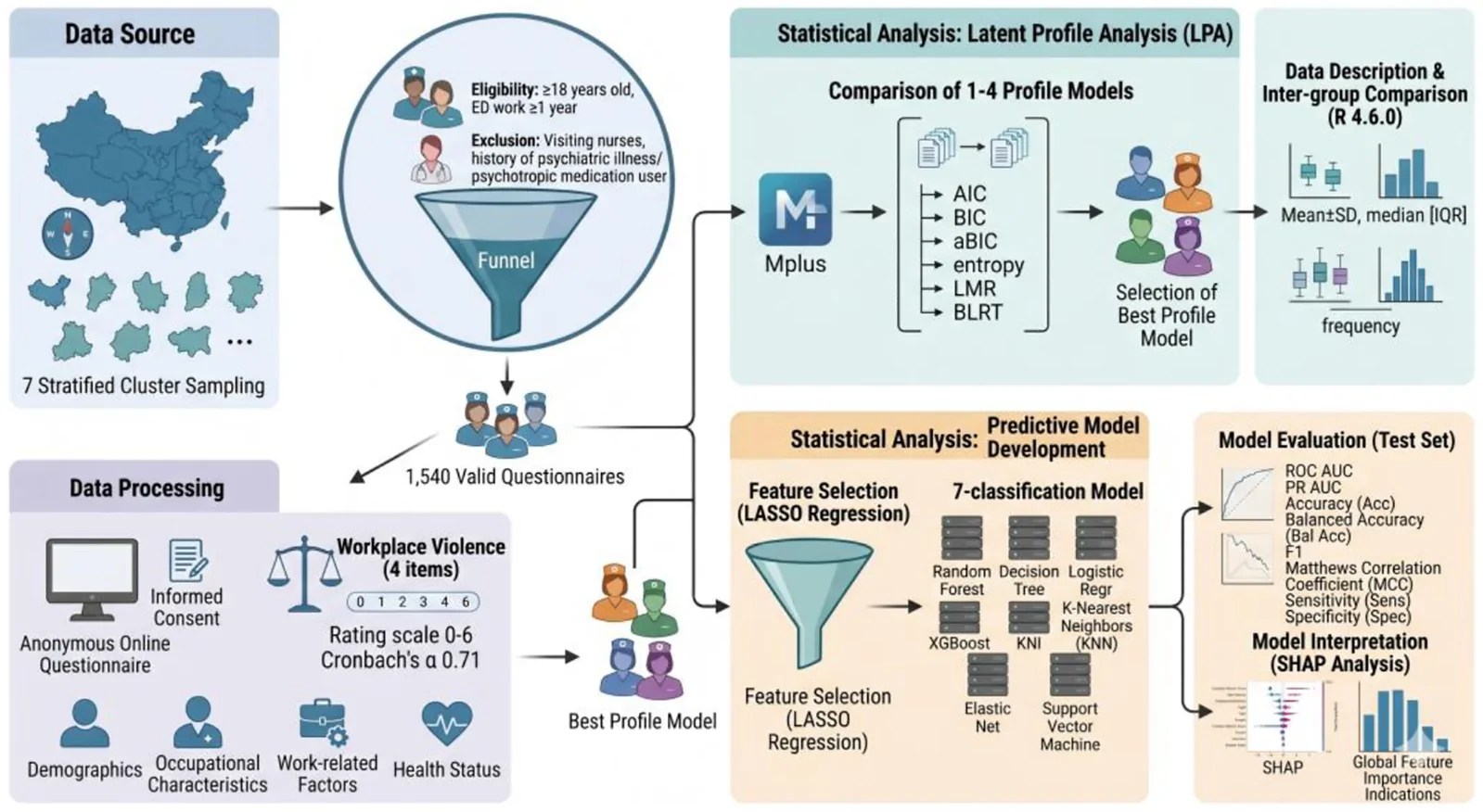
Study workflow for latent profile analysis and machine learning prediction. AIC = Akaike information criterion, BIC = Bayesian information criterion, BLRT = bootstrap likelihood ratio test, IQR = interquartile range, LASSO = least absolute shrinkage and selection operator, LMR = Lo–Mendell–Rubin adjusted likelihood ratio test, LPA = latent profile analysis, PR AUC = area under the precision-recall curve, ROC AUC = area under the receiver operating characteristic curve, SD = standard deviation, SHAP = shapley additive explanations.

Statistical analyses were performed using R (version 4.6.0; R Foundation for Statistical Computing). Continuous variables were summarized as mean ± standard deviation or median with interquartile range, and categorical variables as frequencies and percentages. For predictive modeling, data were stratified by sleep disorder status and randomly divided into training (70%), validation (15%), and test (15%) sets using a fixed seed of 123. Categorical variables were dummy encoded, and numeric variables were median-imputed and normalized. LASSO feature selection and ten-fold cross-validation were performed within the training set. Hyperparameters were tuned using a Latin hypercube search with ROC AUC as the primary metric. The validation set was used for model selection and the test set for final evaluation. Model performance was assessed using ROC AUC, PR AUC, accuracy, balanced accuracy, F1 score, sensitivity, specificity, precision, and Matthews correlation coefficient. SHAP analysis was used to characterize predictor contributions. As a sensitivity analysis, emotional exhaustion, cynicism, and professional efficacy were excluded from the candidate predictor set, and the entire prediction pipeline was repeated using the same data partitioning, feature-selection procedure, and modeling strategy as in the primary analysis. The workflow is shown in Figure 2.

### 2.4. Ethics statement

Ethical approval for this study was obtained from the Ethics Review Committee of West China Hospital, Sichuan University (approval no. 2024.309). All participants provided informed consent before completing the questionnaire. The study was conducted in accordance with the Declaration of Helsinki.

## 3. Results

### 3.1. Demographic characteristics of participants

During the study period, 1551 questionnaires were collected. After excluding 11 questionnaires with identical responses, 1540 participants were included in the final analysis. Participants were recruited from 7 geographical regions of China: North China (9.0%), Northeast China (15.3%), East China (21.6%), Central China (9.0%), South China (8.2%), Southwest China (16.9%), and Northwest China (19.7%). Among the participants, 1211 (78.6%) were female, 1354 (87.9%) had a bachelor’s degree or higher, 963 (62.5%) worked more than 40 hours per week, and 1359 (88.2%) worked night shifts. Of the total sample, 913 (59.3%) screened positive for sleep disorder and 627 (40.7%) screened negative.

Among the 1540 emergency department nurses enrolled in the study, 913 (59.3%) were classified as having sleep disorders and 627 (40.7%) as not having sleep disorders. Significant between-group differences were observed for hospital type, age group, years of work experience, weekly working hours, night shift status, latent workplace violence exposure profile, emotional exhaustion, and cynicism. Compared with nurses without sleep disorders, those with sleep disorders were more likely to work in type 2 hospitals (99.3) and were more frequently aged 30 to 39 years (49.9). Differences were also evident in work experience (*P*=.004, with higher proportions of nurses with sleep disorders having worked for 11 to 15 years (21.5% vs 16.6%) or 16 to 20 years (8.98% vs 6.22%). The sleep disorder group also reported heavier weekly workloads, particularly 49 to 58 h/wk (9.75% vs 5.42%) and ≥59 h/wk (5.04% vs 2.23%; *P* < .001), and a higher prevalence of night shift work (90.1). They also showed higher levels of emotional exhaustion (median [IQR]: 11.0 [7.00–17.0] vs 9.00 [5.00–14.0], *P* < .001) and cynicism (8.00 [4.00–15.0] vs 5.00 [1.00–10.0], *P* < .001), indicating a greater burden of occupational burnout. Details can be seen in Table S1, Supplemental Digital Content 2.

### 3.2. LPA of workplace violence exposure among emergency department nurses

Latent profile analysis was applied to determine whether workplace violence exposure among emergency department nurses clustered into empirically distinguishable subgroups. As reported in Table 1, the fit indices improved as successive profiles were added, a pattern most clearly reflected in the declining AIC, BIC, and aBIC values. The 3-profile solution provided a better fit than the 1- and 2-profile solutions, with an AIC of 16,860.548, a BIC of 16,956.660, and an aBIC of 16,899.479. Its entropy value was 0.980, indicating clear separation of the latent profiles and a high degree of classification accuracy. For this model, both the Lo–Mendell–Rubin adjusted likelihood ratio test and the bootstrap likelihood ratio test were statistically significant (*P* < .001), supporting the superiority of the 3-profile solution over the 2-profile solution. Although the 4-profile model yielded further decreases in the information criteria, the LMR test was not significant (*P* = .162), suggesting that the additional profile did not offer a substantively meaningful improvement in model fit. The 3-profile solution also demonstrated high classification precision. The average posterior probabilities for the moderate multi-form, low-level, and high multi-form violence exposure profiles were 0.970, 0.994, and 0.996, respectively, corresponding to classification uncertainties of 0.030, 0.006, and 0.004. The most likely class assignments included 158 (10.3%), 1341 (87.0%), and 41 (2.7%) participants in the moderate-, low-, and high-exposure profiles, respectively.

**Table 1:** Model fit indices for latent profile models of workplace violence exposure among emergency department nurses.

AIC	BIC	aBIC	Entropy	*P*-value		Latent class probabilities
				LMR	BLRT	
19,932.982	19,975.698	19,950.284	–	–	–	1
17,754.744	17,824.158	17,782.86	0.976	<.001	<.001	0.885/0.115
16,860.548	16,956.660	16,899.479	0.980	<.001	<.001	0.103/0.870/0.027
15,569.714	15,692.523	15,619.458	1.000	.162	<.001	0.0890/0.150/0.736/0.0247

Lower AIC, BIC, and aBIC values indicate better model fit. Higher entropy values indicate greater classification precision. Significant LMR and BLRT *P* values indicate that the *k*-class model fits significantly better than the *k* − 1 class model. Latent class probabilities indicate the estimated proportion of participants in each latent class.

AIC = Akaike information criterion, BIC = Bayesian information criterion, aBIC = sample-size adjusted Bayesian information criterion, LMR = Lo–Mendell–Rubin adjusted likelihood ratio test, BLRT = bootstrap likelihood ratio test.

**Figure 3.**
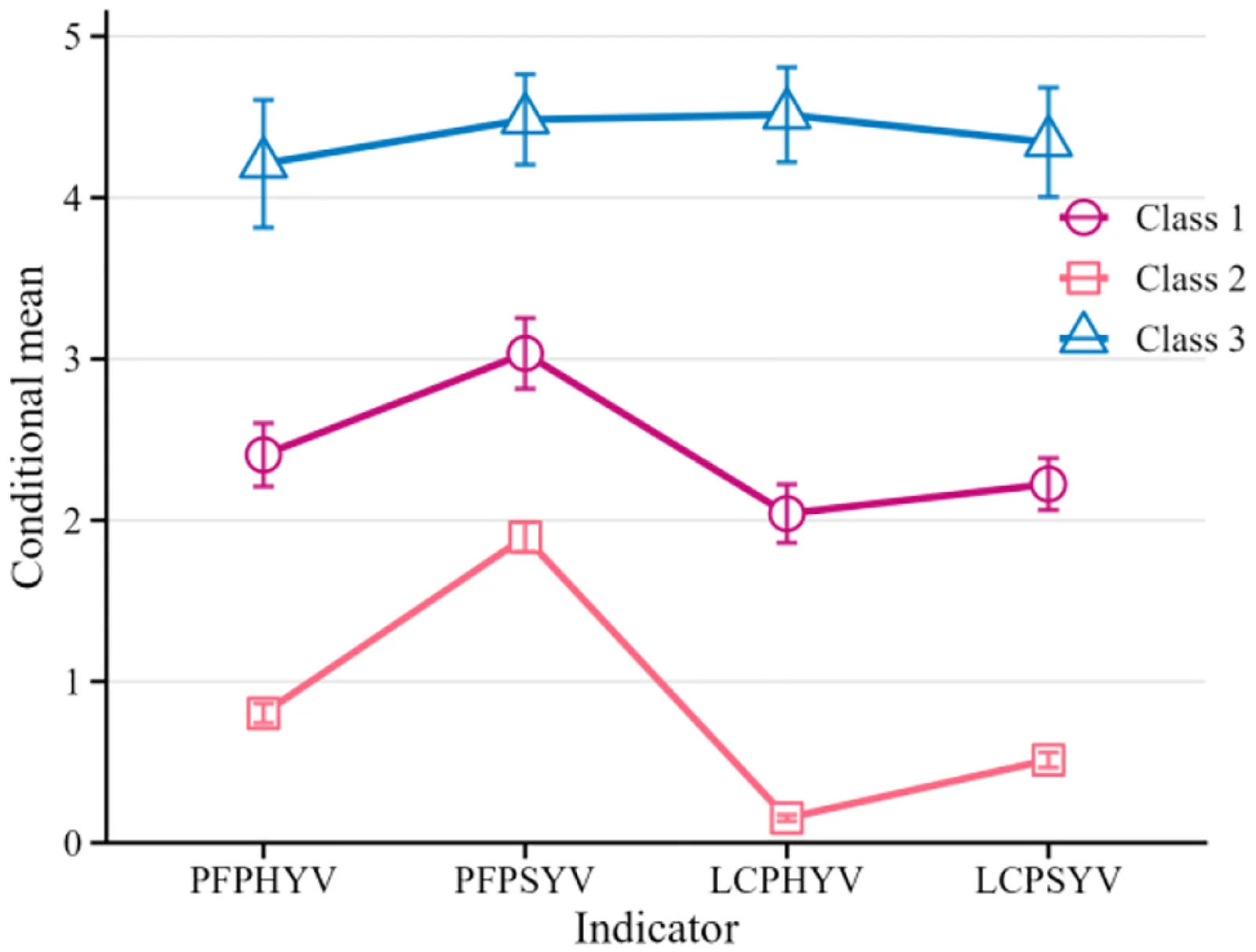
Conditional mean profiles of the 3 latent profile classes across 4 indicators.

Based on the conditional mean distributions observed across the 4 workplace violence indicators – physical violence perpetrated by patients or their family members (PFPHYV), psychological violence perpetrated by patients or their family members (PFPSYV), physical violence perpetrated by leaders or colleagues (LCPHYV), and psychological violence perpetrated by leaders or colleagues (LCPSYV) – the 3 latent profiles were named the low-level violence exposure profile (class 2), the moderate multi-form violence exposure profile (class 1), and the high multi-form violence exposure profile (class 3). The low-level violence exposure profile comprised the largest proportion of participants, accounting for 87.0% of the sample; across all 4 indicators, its conditional means remained consistently low, suggesting relatively limited exposure to workplace violence. The moderate multi-form violence exposure profile included 10.3% of participants and showed an overall intermediate pattern of exposure, although the elevation was more apparent for indicators related to psychological violence. By comparison, the high multi-form violence exposure profile represented only 2.7% of the sample, yet it exhibited persistently high conditional means across all indicators, indicating exposure to multiple forms of workplace violence rather than to a single isolated type (see Fig. 3 and Table S3, Supplemental Digital Content 3).

### 3.3. Machine-learning prediction

#### 3.3.1. LASSO-based feature selection

**Figure 4.**
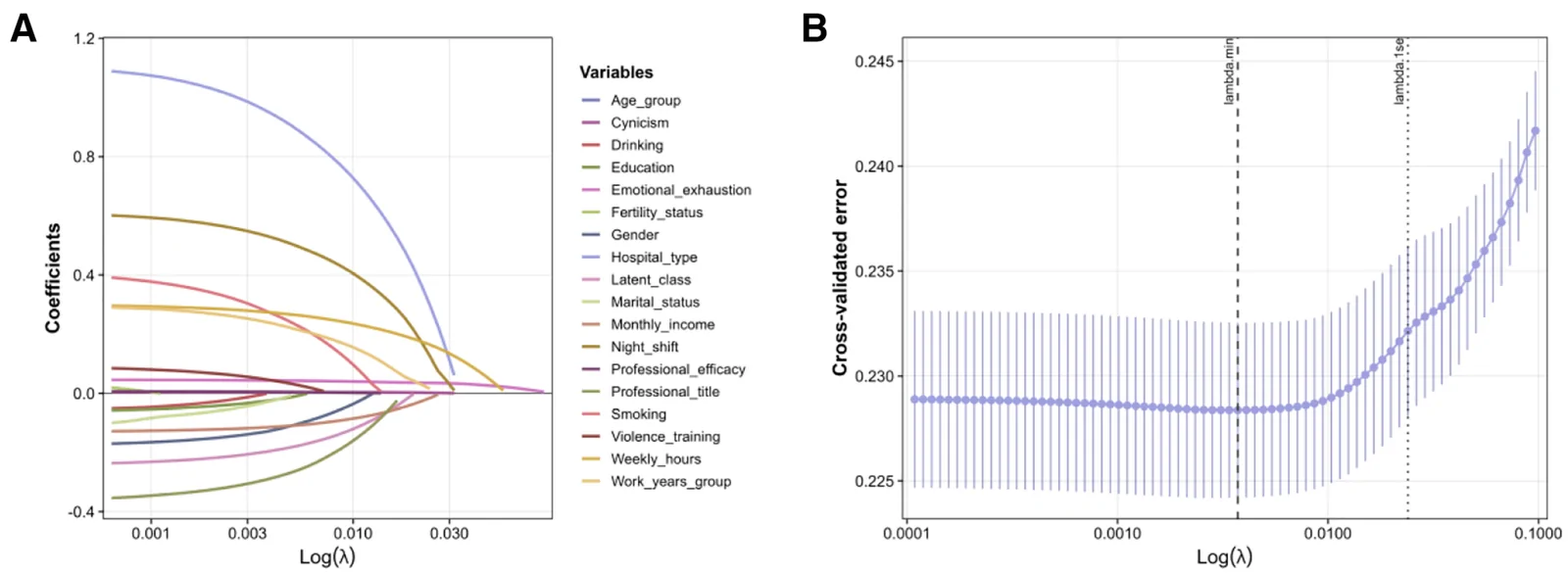
Feature selection using least absolute shrinkage and selection operator regression. (A) Coefficient paths of candidate predictors as the penalty parameter λ increased. (B) Ten-fold cross-validation for determining the optimal λ. Predictors with nonzero coefficients at the selected λ were included in downstream machine learning models.

Least absolute shrinkage and selection operator (LASSO) regression was conducted before model development. As the penalty parameter λ increased, the candidate regression coefficients were steadily drawn toward zero, as depicted in Figure 4; this shrinkage indicated progressively stronger regularization, particularly for predictors carrying weaker signals. The optimal λ was determined by ten-fold cross-validation. Around this selected value, the cross-validation error showed only limited fluctuation, suggesting that the retained variables maintained relatively stable predictive relevance. Predictors with nonzero coefficients at the selected λ were then advanced to the machine learning stage. By using this penalized selection approach, demographic characteristics, occupational factors, workplace violence exposure profiles, and burnout-related indicators could be examined within a single modeling framework, while variables with negligible incremental predictive value were removed. The resulting LASSO-selected predictors served as the shared feature set for all subsequent classification models.

#### 3.3.2. Model performance

**Figure 5.**
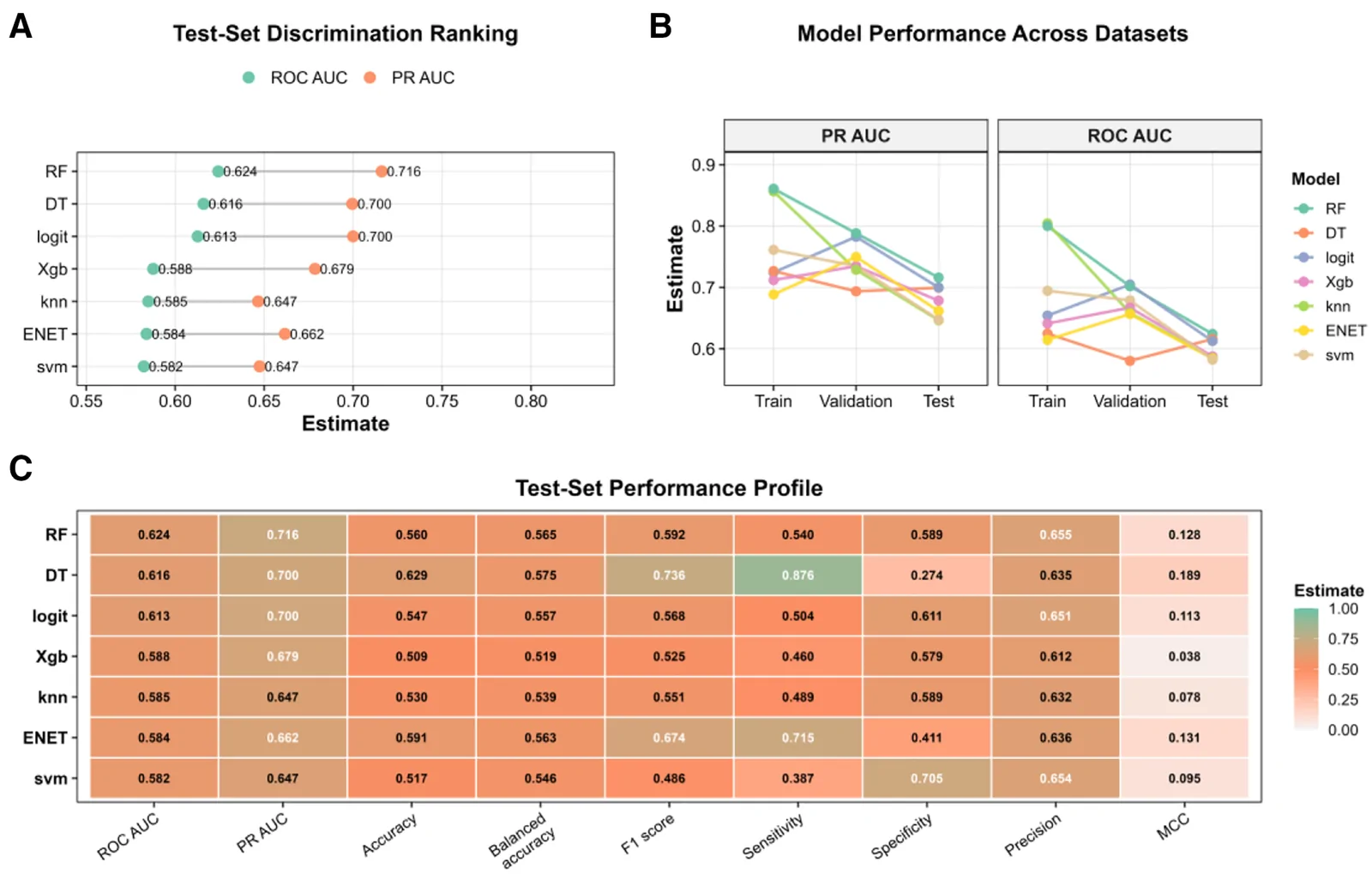
Comparative performance of machine learning models for predicting sleep disorders among emergency department nurses. (A) Test-set discrimination ranking of the 7 models based on ROC AUC and PR AUC. (B) Model performance across the training, validation, and test datasets according to PR AUC and ROC AUC. (C) Test-set performance profile of the 7 models across discrimination and threshold-dependent metrics, including ROC AUC, PR AUC, accuracy, balanced accuracy, F1 score, sensitivity, specificity, precision, and Matthews correlation coefficient. DT = decision tree, ENET = elastic net, KNN = k-nearest neighbors, RF = random forest, SVM = support vector machine, XGB = extreme gradient boosting.

Seven classification algorithms were developed to predict sleep disorder status among emergency department nurses. In the independent test dataset, the random forest model showed the highest, although modest, discrimination, with an ROC AUC of 0.624 (95% CI, 0.551–0.697) and a PR AUC of 0.716 (95% CI, 0.642–0.790). Its Matthews correlation coefficient was 0.128, indicating limited overall classification performance. The decision tree and logistic regression models yielded ROC AUCs of 0.616 and 0.613, respectively, while both achieved a PR AUC of 0.700. Across the remaining models, ROC AUCs ranged from 0.582 to 0.588, with corresponding PR AUCs between 0.647 and 0.679. Detailed results are presented in Figures 5, S1, Supplemental Digital Content 4, and Table S4, Supplemental Digital Content 5.

The calibration curve showed an overall agreement between predicted probabilities and observed event rates, although some deviations were present at lower and higher probability ranges. The confusion matrix indicated that the model correctly classified 56 nurses without sleep disorders and 74 nurses with sleep disorders, while 63 false-positive and 39 false-negative classifications were observed. Decision curve analysis suggested that the model provided positive net benefit across a range of threshold probabilities compared with the treat-none strategy. Detailed results are presented in Figures S2–S4, Supplemental Digital Content 6–8.

#### 3.3.3. Model interpretation

**Figure 6.**
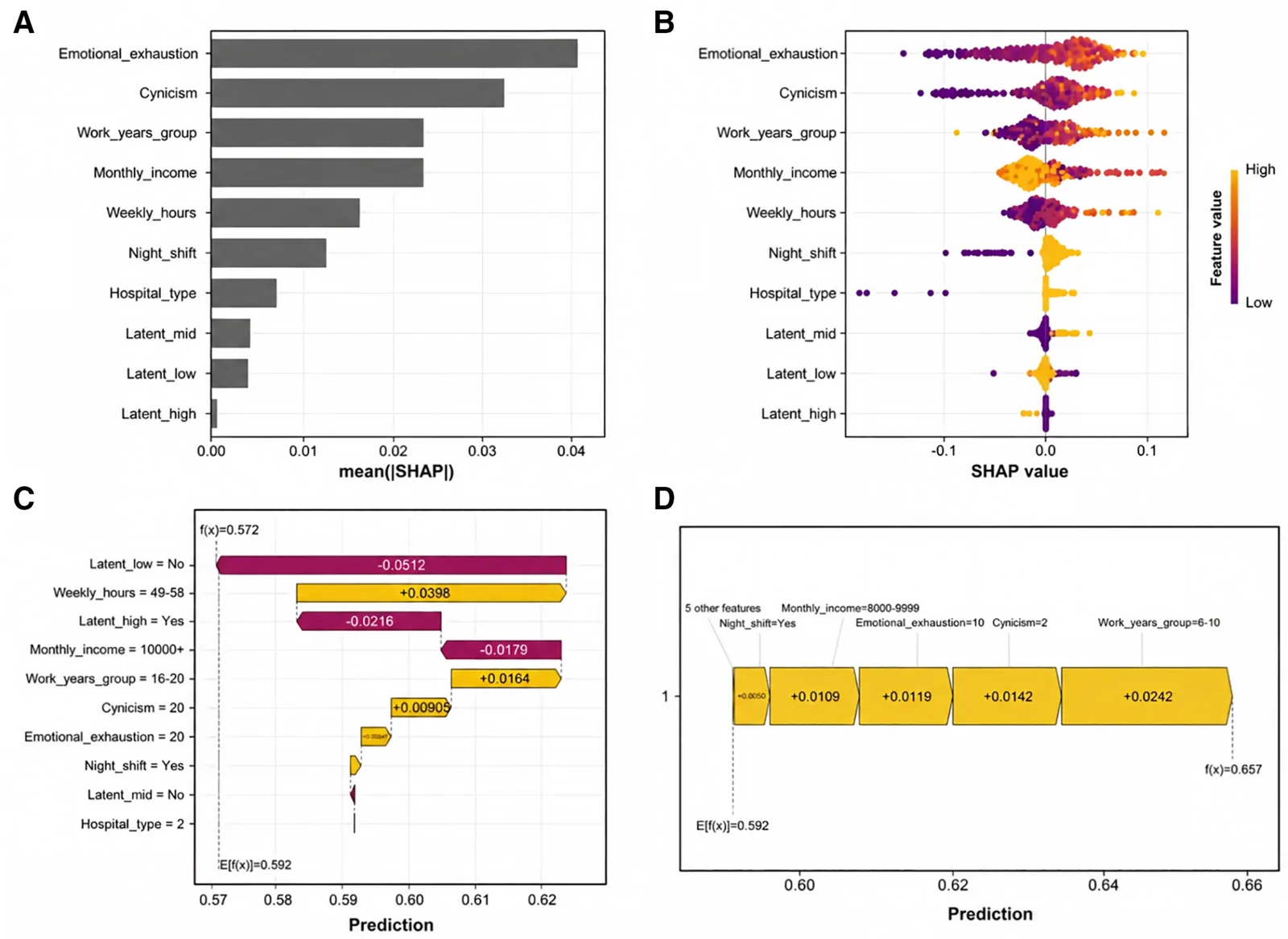
SHAP-based interpretation of feature contributions to random forest predictions. (A) Global feature importance ranked by mean absolute SHAP values. (B) SHAP summary plot showing the distribution of feature effects across observations. (C, D) Individual-level SHAP explanations for 2 representative cases. SHAP = shapley additive explanations.

SHAP analysis was performed to characterize the contribution of predictors in the random forest model. In Figure 6A, predictors are ordered by mean absolute SHAP value. Emotional exhaustion had the largest contribution, followed by cynicism, work years group, monthly income, weekly working hours, and night shift status. Lower mean absolute SHAP values were observed for hospital type and for the latent workplace violence exposure profile variables, including the moderate-, low-, and high-exposure profiles.

Figure 6B displays the distribution of SHAP values across individual observations. Emotional exhaustion and cynicism showed the broadest distributions among the included predictors. Work years group, monthly income, weekly working hours, and night shift status also demonstrated visible dispersion in SHAP values. Hospital type and the latent workplace violence exposure profile variables showed comparatively narrow SHAP value ranges.

Figure 6C, D shows individual-level SHAP explanations for 2 representative cases. In the first case, the baseline value was 0.592, and the final predicted value was 0.572. Positive SHAP contributions were observed for weekly working hours of 49 to 58 hours (+0.0398), work years group of 16 to 20 years (+0.0164), cynicism score of 20 (+0.00905), and emotional exhaustion score of 20. Negative SHAP contributions were observed for Latent_low = No, Latent_high = Yes, and monthly income ≥ 10,000, with values of −0.0512, −0.0216, and −0.0179, respectively.

For the second case, the baseline value was also 0.592, while the final predicted value increased to 0.657. Positive SHAP contributions were recorded for work years group of 6 to 10 years (+0.0242), cynicism score of 2 (+0.0142), emotional exhaustion score of 10 (+0.0119), monthly income of 8000 to 9999 (+0.0109), and night shift work.

## 4. Discussion

This study aimed to explore the latent profile characteristics of workplace violence exposure among emergency department nurses and its association with sleep disorders. Three profiles of workplace violence exposure were distinguished: low-level violence exposure, moderate multi-form violence exposure, and high multi-form violence exposure, comprising 87.0%, 10.3%, and 2.7% of participants, respectively. Sleep disorders were also frequent, affecting 59.3% of the sample. Taken together, these results indicate that workplace violence in emergency nursing is poorly represented by a simple exposed-versus-unexposed framework. Exposure differed across source, form, and intensity, while sleep disorder status was associated with a broader constellation of occupational and psychological characteristics.^[21-24]^

Although the low-level exposure profile included most participants, 13.0% of nurses were classified into profiles marked by more complex or more intense exposure. The moderate multi-form profile was characterized mainly by elevated psychological violence, whereas the high multi-form profile showed sustained elevation across all 4 indicators, including physical and psychological violence from patients or family members and from leaders or colleagues. This pattern suggests that, for a small but meaningful subgroup of emergency department nurses, workplace violence exposure may represent a recurring, multi-source occupational stressor rather than a series of isolated events.^[25,26]^ This interpretation is consistent with broader evidence that workplace violence affects healthcare workers across clinical settings, with verbal and psychological violence especially common in healthcare environments.^[21]^

Sleep disorders were accompanied by greater occupational and psychological strain.^[27–29]^ Nurses with sleep disorders reported longer weekly working hours, more frequent night shift work, and higher burnout-related scores.^[30]^ These associations should be interpreted as concurrent relationships rather than temporal effects. Median emotional exhaustion and cynicism scores were higher in the sleep disorder group than in nurses without sleep disorders, at 11.0 versus 9.00 and 8.00 versus 5.00, respectively. These findings are consistent with evidence linking night shift frequency, occupational stress, and shift-related circadian disruption to sleep problems among nurses.^[31]^ A recent systematic review likewise reported a global prevalence of shift work disorder of 45.5% among nurses and identified stress, night shift frequency, and age as relevant risk factors.^[12]^

A central contribution of this study lies in the SHAP-based interpretation of the random forest model.^[16]^ Although latent violence exposure profiles varied across sleep disorder status, their contributions to model predictions were smaller than those of several burnout-related and occupational characteristics. Emotional exhaustion had the highest mean absolute SHAP value, followed by cynicism, work years group, monthly income, weekly working hours, and night shift status, while hospital type and the latent violence profile variables contributed less to the fitted model.^[14]^ Higher SHAP values for burnout-related and occupational variables therefore should not be interpreted as evidence of greater causal importance, nor should the observed ranking be used to determine intervention priorities.

The machine learning results also suggest that sleep disorder prediction in this population is multidimensional, although the overall performance was modest.^[32,33]^ Among the 7 algorithms, random forest achieved the highest test-set ROC AUC and PR AUC, with values of 0.624 and 0.716, respectively. Decision tree and logistic regression showed slightly lower ROC AUC values of 0.616 and 0.613, and both reached a PR AUC of 0.700. Model selection may therefore need to reflect the intended use, whether broad early screening or more conservative identification of high-risk nurses is prioritized.^[34]^

Mean absolute SHAP values were calculated to characterize the relative contribution of individual predictors to the model output.^[35]^ Emotional exhaustion showed the greatest contribution, followed by cynicism, work years group, monthly income, weekly working hours, and night shift status. The direction of SHAP values further indicated the relationship between each feature and model predictions, with positive values corresponding to higher predicted probabilities of sleep disorders and negative values indicating lower probabilities.^[36]^ Higher burnout-related scores, longer working hours, night shift work, and lower monthly income were associated with increased model-predicted probabilities.^[37,38]^ In contrast, latent violence exposure profiles showed relatively smaller contributions within the fitted model.^[39]^ The high-exposure profile requires cautious interpretation given its limited sample size and unstable SHAP direction. These findings represent feature contributions to prediction rather than causal effects and should not be interpreted as evidence for causal mechanisms or as a basis for prioritizing interventions.^[40,41]^

Several limitations should be acknowledged, including the cross-sectional design, self-reported measures, a small high multi-form violence exposure profile, and lack of external validation. The findings reveal heterogeneous violence exposure patterns and identify burnout-related and occupational characteristics as important predictors of sleep disorder status. Future studies will externally validate the model in independent populations to assess its generalizability.

## 5. Conclusion

This study identified 3 distinct workplace violence exposure profiles among emergency department nurses and found a high prevalence of sleep disorders. Burnout-related symptoms and occupational factors, especially emotional exhaustion, cynicism, workload, and night shift work, were key predictive signals. These findings support profile-specific violence prevention and early sleep risk assessment.

## Acknowledgments

The authors thank all emergency department nurses who participated in this study and acknowledge the assistance of hospital coordinators and administrators involved in participant recruitment and questionnaire administration. Their valuable support contributed to the successful completion of this survey.

## Author contributions

**Conceptualization:** Mei Yuan, Wei Zhang, Le Tong.

**Data curation:** Qingli Chen, Mei Yuan.

**Formal analysis:** Qingli Chen, Mei Yuan.

**Funding acquisition:** Qingli Chen.

**Investigation:** Qingli Chen, Wei Zhang.

**Methodology:** Qingli Chen, Wei Zhang.

**Project administration:** Wei Zhang, Le Tong.

**Software:** Mei Yuan.

**Supervision:** Wei Zhang, Le Tong.

**Validation:** Qingli Chen, Mei Yuan, Wei Zhang, Le Tong.

**Visualization:** Mei Yuan, Le Tong.

**Writing – original draft:** Qingli Chen, Mei Yuan, Wei Zhang.

**Writing – review & editing:** Qingli Chen, Mei Yuan, Wei Zhang, Le Tong.
















