# Supplementary material for: Latent profiles of workplace violence exposure and predictive modeling of sleep disorders among emergency department nurses: A nationwide cross-sectional study
Source: Medicine (Baltimore). 2026 Sep 4;105(36):e50549. doi: 10.1097/MD.0000000000050549 (PMC13549643; doi:10.1097/MD.0000000000050549)
Supplement: Supplementary file 1 [file medi-105-e50549-s001.docx]

**Table S2**. Demographic characteristics of emergency department nurses

| **Variable** | **Sleep_disorder** | | ***p.overall*** |
| --- | --- | --- | --- |
|  | **No(*N=627*)** | **Yes(*N=913*)** |  |
| Hospital_type: |  |  | 0.002 |
| Tertiary specialized hospital | 17 (2.71%) | 6 (0.66%) |  |
| Tertiary general hospital | 610 (97.3%) | 907 (99.3%) |  |
| Age_group: |  |  | 0.006 |
| 20-29 | 261 (41.6%) | 321 (35.2%) |  |
| 30-39 | 278 (44.3%) | 456 (49.9%) |  |
| 40-49 | 65 (10.4%) | 118 (12.9%) |  |
| 50+ | 23 (3.67%) | 18 (1.97%) |  |
| Gender: |  |  | 0.279 |
| Female | 484 (77.2%) | 727 (79.6%) |  |
| Male | 143 (22.8%) | 186 (20.4%) |  |
| Marital_status: |  |  | 0.628 |
| Single | 233 (37.2%) | 327 (35.8%) |  |
| Married | 394 (62.8%) | 586 (64.2%) |  |
| Fertility_status: |  |  | 0.380 |
| No children | 298 (47.5%) | 412 (45.1%) |  |
| Have children | 329 (52.5%) | 501 (54.9%) |  |
| Education: |  |  | 0.902 |
| Associate | 77 (12.3%) | 109 (11.9%) |  |
| Bachelor+ | 550 (87.7%) | 804 (88.1%) |  |
| Professional_title: |  |  | 0.387 |
| Jr | 369 (58.9%) | 538 (58.9%) |  |
| Mid | 229 (36.5%) | 345 (37.8%) |  |
| Sr | 29 (4.63%) | 30 (3.29%) |  |
| Work_years_group: |  |  | 0.004 |
| 0-5 | 228 (36.4%) | 263 (28.8%) |  |
| 6-10 | 197 (31.4%) | 296 (32.4%) |  |
| 11-15 | 104 (16.6%) | 196 (21.5%) |  |
| 16-20 | 39 (6.22%) | 82 (8.98%) |  |
| 20+ | 59 (9.41%) | 76 (8.32%) |  |
| Weekly_hours: |  |  | <0.001 |
| 0-40 | 272 (43.4%) | 305 (33.4%) |  |
| 41-48 | 307 (49.0%) | 473 (51.8%) |  |
| 49-58 | 34 (5.42%) | 89 (9.75%) |  |
| 59+ | 14 (2.23%) | 46 (5.04%) |  |
| Night_shift: |  |  | 0.007 |
| No | 91 (14.5%) | 90 (9.86%) |  |
| Yes | 536 (85.5%) | 823 (90.1%) |  |
| Monthly_income: |  |  | 0.077 |
| 0-4000 | 20 (3.19%) | 37 (4.05%) |  |
| 4000-5999 | 56 (8.93%) | 93 (10.2%) |  |
| 6000-7999 | 86 (13.7%) | 164 (18.0%) |  |
| 8000-9999 | 151 (24.1%) | 217 (23.8%) |  |
| 10000+ | 314 (50.1%) | 402 (44.0%) |  |
| Smoking: |  |  | 0.183 |
| No | 598 (95.4%) | 855 (93.6%) |  |
| Yes | 29 (4.63%) | 58 (6.35%) |  |
| Drinking: |  |  | 1.000 |
| No | 552 (88.0%) | 803 (88.0%) |  |
| Yes | 75 (12.0%) | 110 (12.0%) |  |
| Violence_training | 0.58 (0.49) | 0.58 (0.49) | 0.968 |
| High exposure | 11 (1.75%) | 30 (3.29%) |  |
| Emotional_exhaustion | 9.00 [5.00;14.0] | 11.0 [7.00;17.0] | <0.001 |
| Cynicism | 5.00 [1.00;10.0] | 8.00 [4.00;15.0] | <0.001 |
| Professional_efficacy | 12.0 [6.00;20.0] | 13.0 [6.00;20.0] | 0.183 |
| **Note.** Values are shown as n (%), mean (SD), or median [IQR], as appropriate. p.overall indicates comparisons between nurses with and without sleep disorder. p < 0.05 was considered statistically significant. | | | |
