# Supplementary material for: Latent profiles of workplace violence exposure and predictive modeling of sleep disorders among emergency department nurses: A nationwide cross-sectional study
Source: Medicine (Baltimore). 2026 Sep 4;105(36):e50549. doi: 10.1097/MD.0000000000050549 (PMC13549643; doi:10.1097/MD.0000000000050549)
Supplement: Supplementary file 2 [file medi-105-e50549-s002.docx]

**Table S1.** Codebook of study variables and coding schemes

| **Variable name** | **Description** | **Coding / Values** | **Variable type** |
| --- | --- | --- | --- |
| Sleep_disorder | Sleep disorder status of emergency department nurses | 0 = No; 1 = Yes | Binary categorical outcome |
| Hospital_type | Type of hospital where the nurse worked | 1 = Tertiary specialized hospital; 2 = Tertiary general hospital | Nominal categorical |
| Age_group | Age category of participants | 1 = 20–29; 2 = 30–39; 3 = 40–49; 4 = 50+ | Ordinal categorical |
| Gender | Biological sex of participants | 1 = Female; 2 = Male | Binary categorical |
| Marital_status | Marital status of participants | 1 = Single; 2 = Married | Binary categorical |
| Fertility_status | Childbearing status of participants | 1 = No children; 2 = Have children | Binary categorical |
| Education | Highest educational attainment | 1 = Associate degree; 2 = Bachelor degree or above | Ordinal categorical |
| Professional_title | Professional title or rank of nurses | 1 = Junior; 2 = Middle; 3 = Senior | Ordinal categorical |
| Work_years_group | Years of work experience in nursing or emergency nursing | 1 = 0–5; 2 = 6–10; 3 = 11–15; 4 = 16–20; 5 = >20 years | Ordinal categorical |
| Weekly_hours | Average weekly working hours | 1 = 0–40; 2 = 41–48; 3 = 49–58; 4 = ≥59 hours/week | Ordinal categorical |
| Night_shift | Whether the nurse worked night shifts | 0 = No; 1 = Yes | Binary categorical |
| Monthly_income | Monthly income level | 1 = 0–4000; 2 = 4000–5999; 3 = 6000–7999; 4 = 8000–9999; 5 = ≥10000 CNY/month | Ordinal categorical |
| Smoking | Current smoking status | 0 = No; 1 = Yes | Binary categorical |
| Drinking | Current drinking status | 0 = No; 1 = Yes | Binary categorical |
| Violence_training | Whether the nurse had received workplace violence-related training | 0 = No; 1 = Yes | Binary categorical |
| PFPHYV | Physical violence perpetrated by patients or their family members | 0 = No; 1 = Yes | Binary categorical |
| PFPSYV | Psychological violence perpetrated by patients or their family members | 0 = No; 1 = Yes | Binary categorical |
| LCPHYV | Physical violence perpetrated by leaders or colleagues | 0 = No; 1 = Yes | Binary categorical |
| LCPSYV | Psychological violence perpetrated by leaders or colleagues | 0 = No; 1 = Yes | Binary categorical |
| Emotional_exhaustion | Emotional exhaustion score; higher scores indicate greater emotional exhaustion | Continuous score | Continuous variable |
| Cynicism | Cynicism score; higher scores indicate greater cynicism | Continuous score | Continuous variable |
| Professional_efficacy | Professional efficacy score; higher scores indicate greater professional efficacy | Continuous score | Continuous variable |
