# Supplementary material for: Latent profiles of workplace violence exposure and predictive modeling of sleep disorders among emergency department nurses: A nationwide cross-sectional study
Source: Medicine (Baltimore). 2026 Sep 4;105(36):e50549. doi: 10.1097/MD.0000000000050549 (PMC13549643; doi:10.1097/MD.0000000000050549)
Supplement: Supplementary file 3 [file medi-105-e50549-s003.docx]

**Table S3.** Classification quality and characteristics of the selected three-profile solution

| **Characteristic** | **Moderate multi-form violence exposure** | **Low-level violence exposure** | **High multi-form violence exposure** |
| --- | --- | --- | --- |
| Most likely class membership, n (%) | 158 (10.3) | 1,341 (87.1) | 41 (2.7) |
| Estimated class proportion, % | 10.46 | 86.88 | 2.66 |
| PFPHYV, mean (SE) | 2.405 (0.100) | 0.803 (0.031) | 4.210 (0.202) |
| PFPSYV, mean (SE) | 3.034 (0.112) | 1.896 (0.047) | 4.484 (0.143) |
| LCPHYV, mean (SE) | 2.041 (0.092) | 0.154 (0.010) | 4.514 (0.150) |
| LCPSYV, mean (SE) | 2.224 (0.082) | 0.514 (0.024) | 4.343 (0.173) |
| Average posterior probability (AvePP) | 0.97 | 0.994 | 0.996 |
| Classification uncertainty, 1 − AvePP | 0.03 | 0.006 | 0.004 |
| Posterior probability of Moderate profile | 0.97 | 0.006 | 0.004 |
| Posterior probability of Low profile | 0.029 | 0.994 | 0 |
| Posterior probability of High profile | 0.001 | 0 | 0.996 |

**Note.** Overall entropy for the three-profile solution was 0.980. AvePP indicates the average posterior probability for the most likely latent profile membership. Classification uncertainty was calculated as 1 − AvePP. PFPHYV = physical violence perpetrated by patients or family members; PFPSYV = psychological violence perpetrated by patients or family members; LCPHYV = physical violence perpetrated by leaders or colleagues; LCPSYV = psychological violence perpetrated by leaders or colleagues.
