# Supplementary material for: Latent profiles of workplace violence exposure and predictive modeling of sleep disorders among emergency department nurses: A nationwide cross-sectional study
Source: Medicine (Baltimore). 2026 Sep 4;105(36):e50549. doi: 10.1097/MD.0000000000050549 (PMC13549643; doi:10.1097/MD.0000000000050549)
Supplement: Supplementary file 4 [file medi-105-e50549-s005.docx]

**Table S4.** Sensitivity analysis of model performance after excluding burnout-related variables

| **Performance metric** | **Full model** | **Burnout-excluded model** | **Δ**D |
| --- | --- | --- | --- |
| ROC AUC | 0.624 | 0.603 | −0.021 |
| PR AUC | 0.716 | 0.694 | −0.022 |
| Accuracy | 0.56 | 0.548 | −0.012 |
| Balanced accuracy | 0.565 | 0.55 | −0.015 |
| Sensitivity | 0.54 | 0.518 | −0.022 |
| Specificity | 0.589 | 0.582 | −0.007 |
| F1 score | 0.592 | 0.573 | −0.019 |
| Matthews correlation coefficient | 0.128 | 0.101 | −0.027 |
